# Benzyl Isothiocyanate, a Vegetable-Derived Compound, Induces Apoptosis via ROS Accumulation and DNA Damage in Canine Lymphoma and Leukemia Cells

**DOI:** 10.3390/ijms222111772

**Published:** 2021-10-29

**Authors:** Marta Henklewska, Aleksandra Pawlak, Rong-Fang Li, Jine Yi, Iwona Zbyryt, Bożena Obmińska-Mrukowicz

**Affiliations:** 1 Department of Pharmacology and Toxicology, Wrocław University of Environmental and Life Sciences, C.K. Norwida 31, 50-375 Wrocław, Poland; aleksandra.pawlak@upwr.edu.pl (A.P.); b.mrukowicz@gmail.com (B.O.-M.); 2 Hunan Engineering Research Center of Livestock and Poultry Health Care, Colleges of Veterinary Medicine, Hunan Agricultural University, Changsha 410128, China; lrf0408@126.com (R.-F.L.); yibinzhen@163.com (J.Y.); 3 Department of Epizootiology and Clinic of Birds and Exotic Animals, Wrocław University of Environmental and Life Sciences, C.K. Norwida 31, 50-375 Wrocław, Poland; iwona.zbyryt@upwr.edu.pl

**Keywords:** BITC, anti-cancer drugs, canine cancers, cancer treatment, oxidative stress

## Abstract

Treatment of neoplastic diseases in companion animals is one of the most important problems of modern veterinary medicine. Given the growing interest in substances of natural origin as potential anti-cancer drugs, our goal was to examine the effectiveness of benzyl isothiocyanate (BITC), a compound found in cruciferous vegetables, against canine lymphoma and leukemia. These are the one of the most common canine cancer types, and chemotherapy is the only treatment option. The study involved established cell lines originating from various hematopoietic malignancies: CLBL-1, GL-1, CLB70 and CNK-89, immortalized noncancerous cell lines: MDCK and NIH-3T3 and canine peripheral blood mononuclear cells (PBMCs). The cytotoxic activity of BITC, apoptosis induction, caspase activity and ROS generation were evaluated by flow cytometry. H2AX phosphorylation was assessed by western blot. The study showed that the compound was especially active against B lymphocyte-derived malignant cells. Their death resulted from caspase-dependent apoptosis. BITC induced ROS accumulation, and glutathione precursor N-acetyl-l-cysteine reversed the effect of the compound, thus proving the role of oxidative stress in BITC activity. In addition, exposure to the compound induced DNA damage in the tested cells. This is the first study that provides information on the activity of BITC in canine hematopoietic malignancies and suggests that the compound may be particularly useful in B-cell neoplasms treatment.

## 1. Introduction

Similarly as in humans, cancer in companion animals is nowadays regarded one of the most important chronic diseases, significantly impacting the quality of life and ultimately leading to its shortening [1]. It is estimated that annual incidence rates of malignant tumors in dogs range from about 100 to over 800 per 100,000 dogs depending on the study. Cancer is the most common cause of death of purebred dogs in the UK, accounting for 27% of deaths, and exceeding 50% in the case of some breeds [2]. Among various types of cancer, neoplasms of the hematopoietic system are responsible for 8–9% of all diagnosed cancer cases, with lymphomas being the most frequent [3]. Non-Hodgkin’s lymphoma was found to be the second most frequently diagnosed cancer after mammary cancer in bitches and the most frequent in male dogs in the studies of Merlo et al. [4]. As in humans, treatment of cancer in companion animals involves surgery, radiotherapy, as well as chemotherapy, which in the case of systemic malignancies, such as neoplasms of hematopoietic origin, may be the only treatment option [5]. The most common treatment modalities utilized in canine lymphoma and leukemia therapy are based on chemotherapeutic protocols applied in human medicine and include anti-cancer drugs belonging to alkylating agents, antimetabolites, anthracyclines and alkaloids derived from *Vinca rosea* [6]. Nevertheless, the therapy based on classical genotoxic agents is often burdened with serious side effects and the appearance of drug resistance, which frequently leads to the treatment failure. One of the methods increasing the treatment efficacy and reducing its toxicity is combining drugs with different molecular targets to obtain a synergistic effect [7]. In the search of new, effective anti-cancer agents, which could be used in combination with a conventional therapy, increasing attention has been focused on substances produced by living organisms, including e.g., edible plants. Nature is a rich source of biologically active substances that have, inter alia, properties that limit the growth of cancer cells and lead to their apoptosis [8]. One such group of phytochemicals with a potential anti-cancer activity, are isothiocyanates (ITCs), found in vegetables from *Cruciferae* family, e.g., cabbage, broccoli, cauliflower or brussels sprouts. ITCs are localized in the cytoplasm bound to glucose in glucosinolates and released via hydrolysis by an extracellular enzyme myrosinase, when a plant is damaged, e.g., during chewing or cooking [9]. These natural constituents of cruciferous vegetables have aroused scientific interest many years ago due to their chemopreventive abilities [10,11,12]. ITCs were proven to inhibit carcinogen activation by cytochrome P-450 isozymes and increase carcinogen detoxification and excretion [13,14]. However, what is the most important from the point of view of their use as potential anti-cancer drugs, they also exhibit direct cytotoxic and proapoptotic activity against cancer cells, including those originating from the hematopoietic system [15,16,17,18]. The mechanism of their action is not fully clarified, nevertheless it is known that ITCs as electrophiles react with nucleophiles, especially those with thiol residues. Rapid reaction of ITCs with glutathione, the most abundant intracellular thiol compound, contributes to the reduction of its concentration in the cell and subsequently increases ROS accumulation. In addition, further growth in ROS concentration is the result of their leakage from the mitochondria due to their oxidative damage caused by ITCs [19,20]. The compounds of this group were shown to inhibit cell proliferation and DNA replication, disturb cell cycle progression and generate DNA damage, what can also account for their selective activity towards cancer cells, since they often present an impaired DNA damage repair response [21]. Moreover, ITCs were shown to promote phosphorylation of stress-activated kinases such as JNK, p38, ERK 1/2 and to induce caspase dependent apoptosis [22,23].

One of the ITCs with confirmed cancer growth-inhibiting activity is benzyl isothiocyanate (BITC). Numerous studies in an in vitro model of human cancers indicated BITC as an agent capable of decreasing cell viability and induce apoptosis in cancer cells [9,17,18,19]. Several pathways were shown to be involved in its proapoptotic activity, e.g., loss of mitochondrial potential [17], nuclear accumulation of apoptosis-inducing factor (AIF), downregulation of myeloid cell leukemia-1 (Mcl-1), translocation of Bcl2-associated X protein (BAX) and cytochrome C release from mitochondria [18], as well as initiation of caspase activation [17,18]. The agent is also capable of arresting the cell cycle in G2/M phase [9,18] and stimulate differentiation of cancer cells [17]. In addition, BITC inhibited the growth of leukemia Jurkat xenografts in nude mouse [18].

However, although anti-cancer activity of the compound was confirmed in numerous studies on human cancers, its activity against canine neoplasms has not been investigated so far. Therefore, to assess the possibility of introducing ITCs into companion animal oncology, we decided to preliminarily evaluate the cytotoxic and proapoptotic activity of BITC against the cancers of the hematopoietic system in dogs. The aim of the presented study was to examine selectivity of the compound toward canine lymphoma and leukemia cells and to elucidate the mechanisms leading to cell death after exposure to BITC.

## 2. Results

### 2.1. BITC Exerts a Cytotoxic Effect on Canine Lymphoma and Leukemia Cell Lines and Cells Derived from B Lymphocytes Are Particularly Sensitive to the Compound

**Figure 1:**
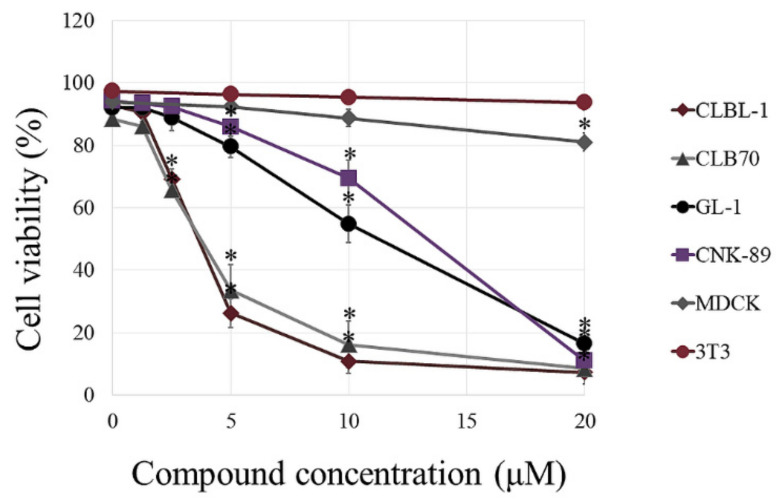
Comparison of the cytotoxic effect of benzyl isothiocyanate (BITC) on noncancerous and canine cancer cell lines. The cells were exposed to increasing concentration of BITC for 24 h. Cell viability was assessed by cytometric analysis following propidium iodide (PI) staining. The values are means ± SD of three independent experiments. * Considered significant in comparison with control (*p* < 0.05).

**Figure 2:**
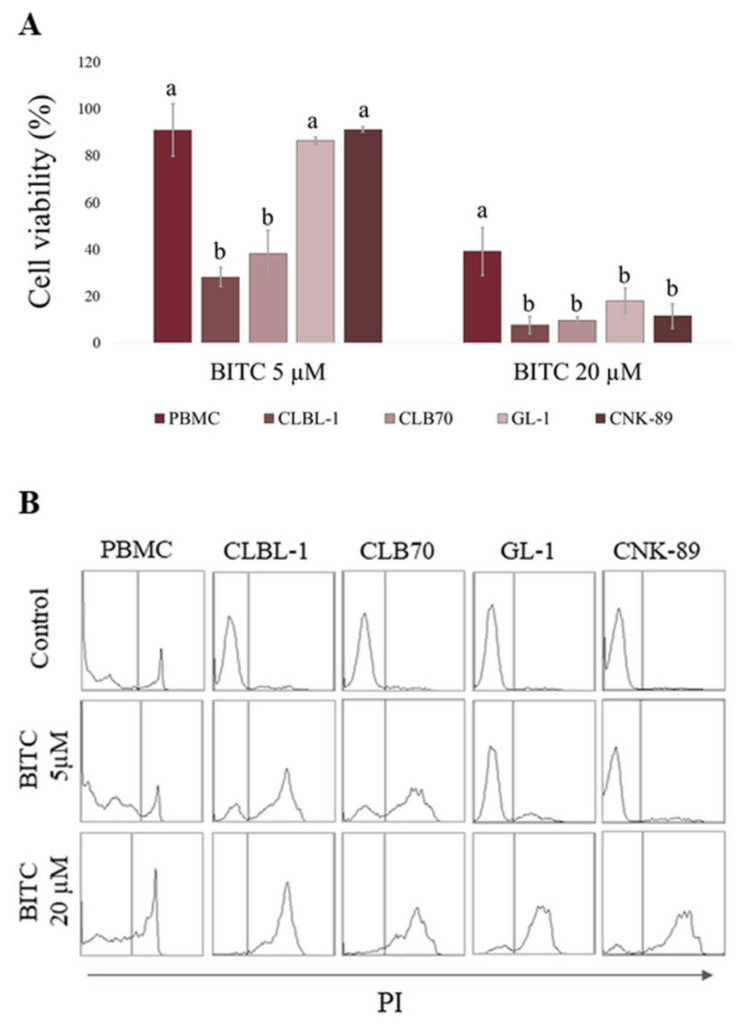
Comparison of BITC activity in canine peripheral blood mononuclear cells (PBMCs) and canine hematopoietic cancer cells (CLBL-1, CLB70, GL-1 and CNK-89) after 24 h incubation with BITC. (**A**) The number of viable cells in BITC treated groups was calculated as a percentage of the viability of untreated control cells. ^a,b^ Values with different letters in the superscript differ statistically within the same treatment group (*p* < 0.05). (**B**) Representative histograms of cells stained with PI.

At the first step of the research, we assessed the cytotoxic effect of the compound in canine hematopoietic cancer cells and compared it to the effect exerted on noncancerous cells. BITC showed concentration-dependent cytotoxic activity against the examined cancer cell lines (Figure 1 and Table S1). What is the most important, the canine hematopoietic cancer cells were more sensitive to BITC than noncancerous cells. The difference was the most pronounced when comparing the sensitivity of the established cell lines. The concentration of 20 µM that killed almost 100% of cells in all cancer cell lines, reduced the viability of canine kidney cells (MDCK cell line) by only about 19%. The murine fibroblasts (NIH 3T3 cell line) were even more resistant and their viability was not changed in a statistically significant manner at this concentration in comparison to the control (*p* > 0.05). Among the cancer cell lines, the most sensitive to BITC were those derived from B lymphocytes: CLBL-1 and CLB70 with EC_50_ 3.63 ± 0.21 µM and 3.78 ± 0.21 µM, respectively. CNK-89 cell line (NK-cell lymphoma) was the most resistant with EC_50_ 13.33 ± 0.93 µM (Table 1). Two the most sensitive cell lines also showed the greatest differences in their sensitivity as compared with peripheral blood mononuclear cells (PBMCs) isolated from blood of healthy dogs. Incubation with 5 µM BITC for 24 h reduced the number of viable CLBL-1 and CLB70 cells to 28.14 ± 4.06 and 38.11 ± 9.97% relative to the untreated cells, while in the case of PBMCs this value was 91.00 ± 11.14%. More resistant GL-1 and CNK-89 cells had, at this concentration, a similar survival rate to PBMCs (*p* > 0.05). Nevertheless, the highest concentration used in the study (20 µM) was more potent toward all canine cancer cell lines than toward PBMCs (*p* < 0.05) (Figure 2 and Table S2).

### 2.2. BITC-Mediated Death Results from Caspases-Dependent Apoptosis in CLB70 and CLBL-1 Cells That Can Be Abolished by Increasing Glutathione Level in the Cells

**Figure 3:**
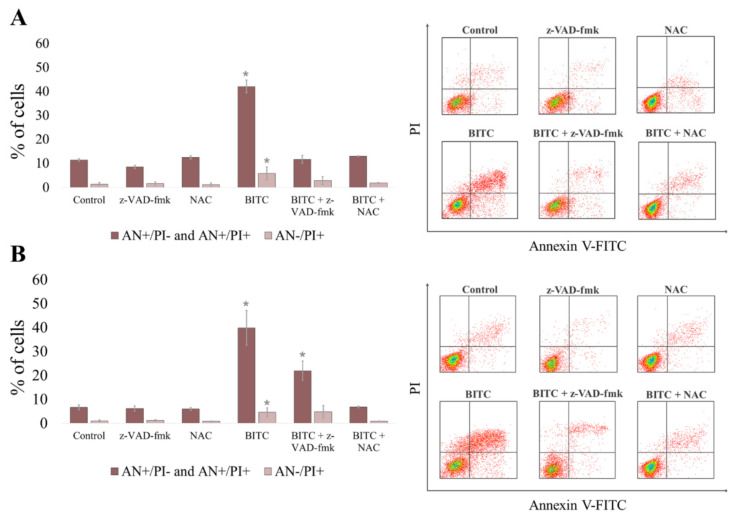
Apoptosis induced by BITC in CLB70 and CLBL-1 cell lines. CLB70 (**A**) and CLBL-1 (**B**) cells were incubated for 16 h with BITC (4 μM) and the number of cells stained with Annexin V-FITC/PI was measured in flow cytometric analysis. The values are means ± SD of three independent experiments. * Considered significant in comparison with control (*p* < 0.05).

In the next step, to confirm that the cell death was due to apoptosis, we investigated externalization of phosphatidylserine on the outer cell membrane of CLB70 and CLBL-1 cells exposed to the drug. After 16 h incubation with BITC at the concentration about EC_50_ for both cell lines, we detected a comparable increase (*p* < 0.05) in the number of apoptotic cells (stained only with Annexin V-FITC for early apoptotic cells and double stained with Annexin-FITC and PI for late apoptotic cells) (Figure 3 and Table S3). In CLBL-1 cell line the amounted to 39.75 ± 7.25%, while in CLB70 it was 42.06 ± 7.44%. In addition, a slight but significant (*p* < 0.05) increase in the number of cells stained only with PI was noticed, which is considered an indicator of necrosis [24]. Both of these effects were reverted by preincubation of the cells with pancaspase inhibitor z-VAD-fmk. This confirmed the engagement of caspases in the apoptosis induced by the compound and suggested that observed single staining with PI was rather the late consequence of caspase activation and apoptosis induction, than primary necrotic activity of BITC. Although in the case of CLBL-1 cell line preincubated with caspase inhibitor the level of apoptosis was still higher than in the control (*p* < 0.05), it can be explained by the fact that in this cell line the inhibitor was unable to completely abolish caspase 3/7 activity (Figure S1 and Table S6). To evaluate the influence of glutathione increase on the effects of BITC, we additionally performed a similar analysis in the cells preincubated with N-acetyl-l-cysteine (NAC)–a glutathione precursor. NAC was capable of reducing apoptosis to the level similar to control cells (*p* > 0.05), which confirmed the protective effect of elevated levels of glutathione on BITC action in the cells.

**Figure 4:**
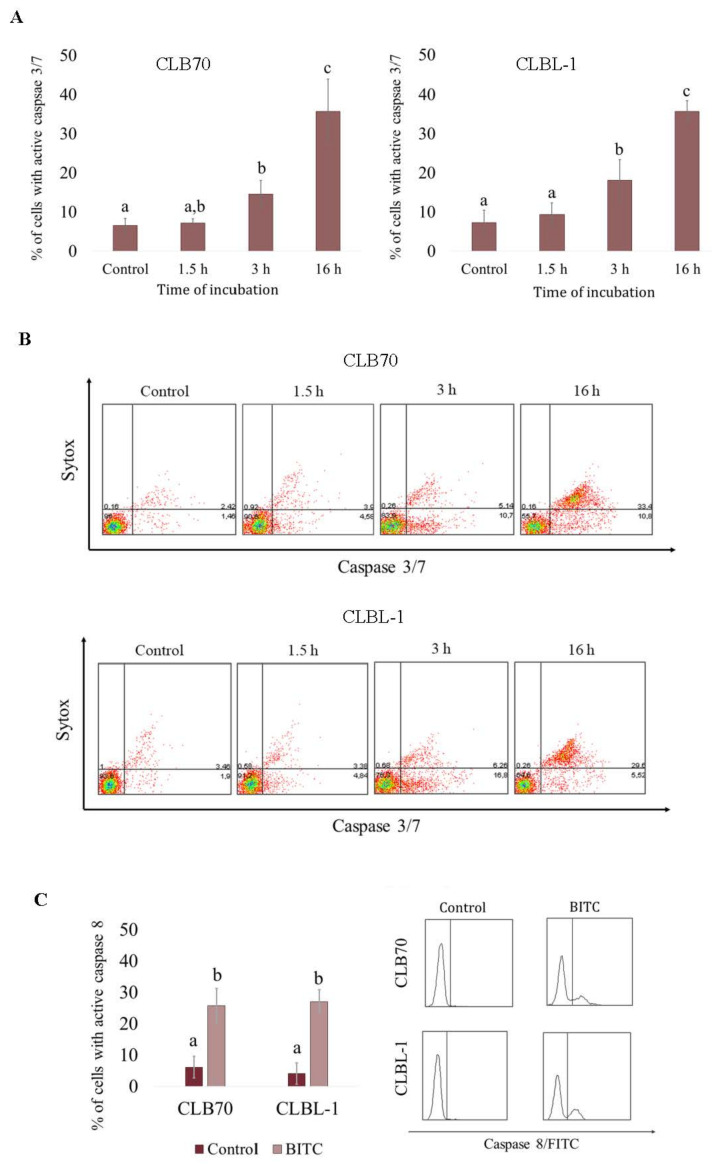
Activation of caspases in CLB70 and CLBL-1 cells after treatment with BITC. (**A**) Activation of caspase 3/7 in CLB70 and CLBL-1 cell lines was investigated in the cells incubated with BITC (4 μM) for 1.5, 3 or 16 h. The number of cells with active caspase 3/7 was evaluated in flow cytometric analysis. (**B**) Representative dotplots showing cells double stained with CellEvent^®^Caspase-3/7 Green Detection Reagent and SYTOX^®^AADvanced™. (**C**) Percentage of cells with active caspase 8. Activation of caspase 8 was estimated in the cytometric analysis of cells stained with FITC-IETD-fmk after 16 h treatment with the compound. The values are means ± SD of three independent experiments. ^a,b,c^ Values with different letters in the superscript differ statistically (*p* < 0.05).

To further assess the role of caspases in the apoptosis induced by BITC, and to determine the time of apoptosis occurrence after exposure to the compound, we evaluated the activation of caspase 3/7. As shown in Figure 4A, 1.5 h exposure to BITC led to a slight, statistically irrelevant (*p* > 0.05), increase in caspase 3/7 activation, while after 3 h, the activation of caspase 3/7 was quite pronounced (*p* < 0.05). Specifically, the number of cells with active caspase 3/7 reached 14.57 ± 3.55% in CLB70 cell line and 18.09 ± 5.31% in CLBL-1 cell line. After 16 h incubation, the numbers rose to 35.77 ± 8.26% in CLB70 cell line and 35.72 ± 2.76% in CLBL-1 cell line (Table S4). Additionally, after 3 h incubation the majority of cells with active caspase 3/7 were negative for Sytox staining, indicating an early stage in the apoptotic process at this time point, while after 16 h the cells were positive for both dyes (Figure 3B). Moreover, 16 h exposure to BITC at the concentration of 4 µM boosted the number of cells with active caspase 8 to 25.77 ± 5.59% and 27.10 ± 3.73% in CLB70 and CLBL-1 cell line, respectively (Figure 4C and Table S5).

### 2.3. BITC Treatment Induces DNA Damage in CLB70 and CLBL-1 Cells

**Figure 5:**
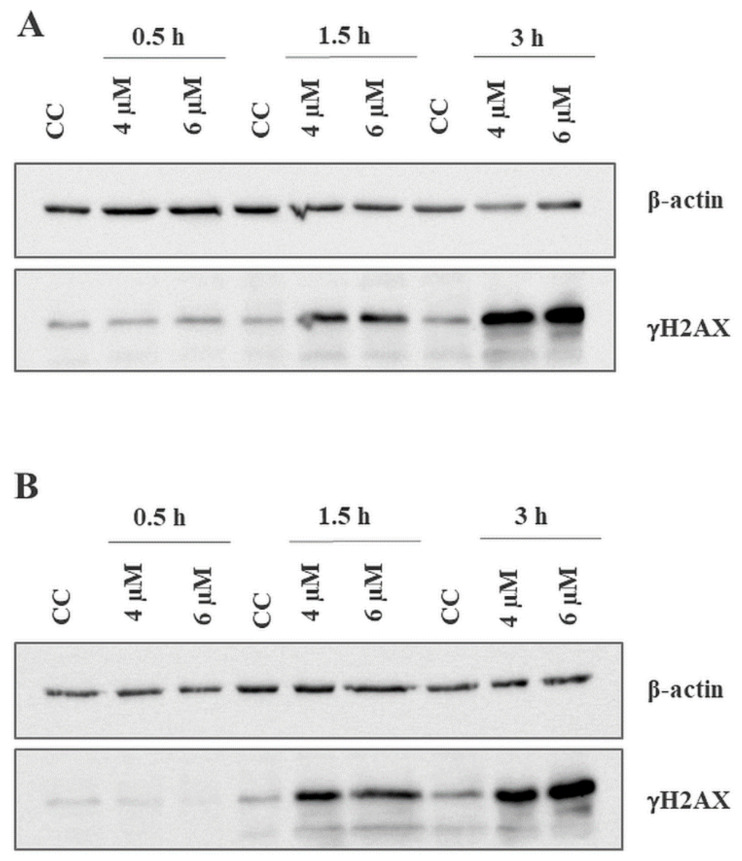
DNA damage generated by BITC in CLB70 and CLBL-1 cells. Increase in the amount of phosphorylated H2AX was examined in CLB70 (**A**) and CLBL-1 (**B**) cells treated with BITC for 0.5, 1.5 or 3 h by western blot analysis.

Since γH2AX is an accepted marker of DNA damage [25], we measured its levels after incubation with the compound for 0.5, 1.5 and 3 h (Figure 5). Thirty minutes of incubation did not result in visible increase in the amount of phosphorylated H2AX, while after 1.5 h and 3 h its spike was considerable in both cell lines and studied concentrations. Most importantly this result, combined with caspase 3/7 activation test showing no apoptosis initiation after 1.5 h, excluded the possibility of DNA damage and H2AX phosphorylation being the results of the apoptosis.

### 2.4. BITC Induces ROS Accumulation in CLB70 and CLBL-1 Cells

**Figure 6:**
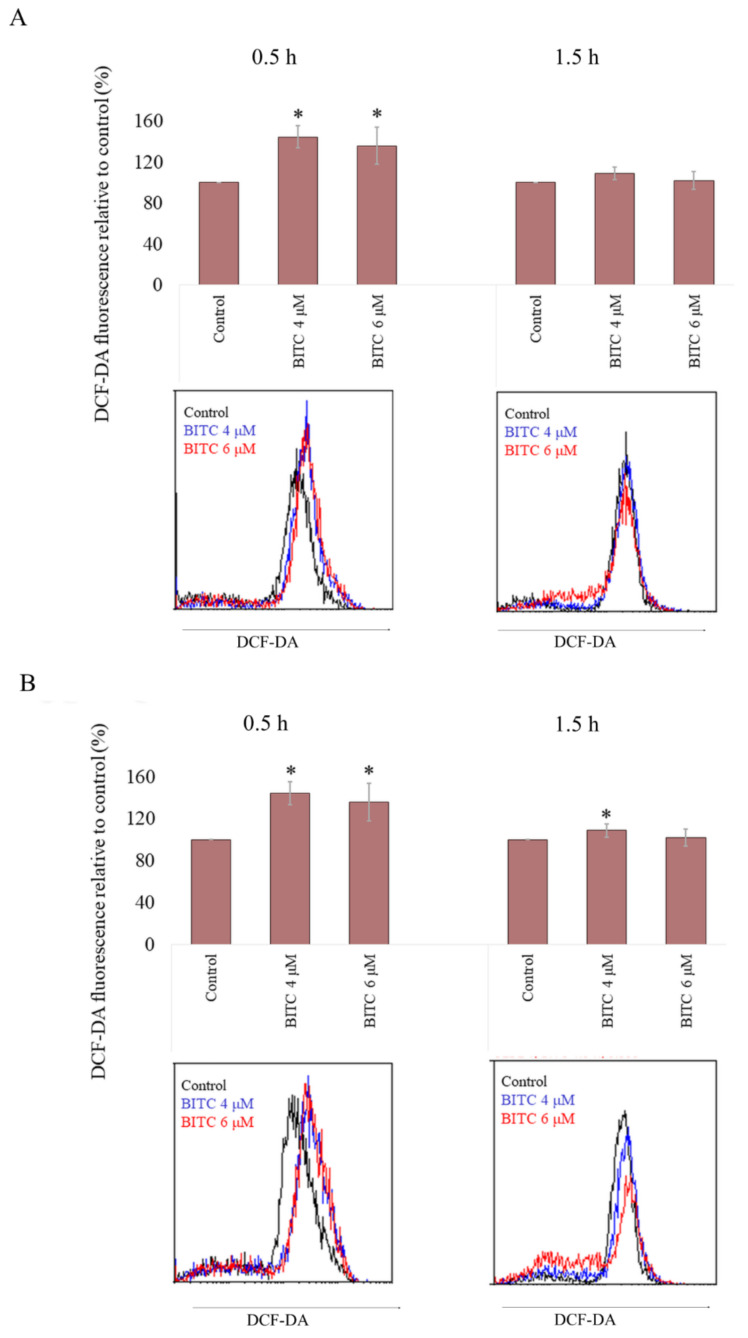
ROS increase after incubation with BITC. 2′,7′-Dichlorofluorescin Diacetate (DCF-DA) fluorescence was measured after incubation of CLB70 (**A**) and CLBL-1 (**B**) cells with BITC for 0.5 h or 1.5 h. The values are means ± SD of three independent experiments. * Considered significant in comparison with control (*p* < 0.05).

To evaluate BITC-generated oxidative stress in CLB70 and CLBL-1 cell lines, the level of ROS was investigated 0.5 h and 1.5 h after exposure to the compound. As after the longer incubation time, apoptosis already occurs in the cells (as demonstrated in the caspase activation test), and may lead to ROS generation [26], the level of ROS was only assessed for the shorter incubation time. ROS concentration was compared with the basal level in untreated cells, in which it was assumed to be 100%. After 0.5 h incubation with 4 μM of BITC, ROS concentration reached 144.36 ± 10.89% and 152.33 ± 13.40% in CLB70 and CLBL-1 cell lines, respectively. This effect was concentration independent, as it remained at a similar level after incubation with 6 μM of BITC (Figure 6 and Table S7). Surprisingly, after 1.5 h incubation with BITC, ROS concentration decreased to the almost basal level and only in CLBL-1 cell line it remained statistically different from the control (*p* < 0.05).

## 3. Discussion

In the search for more effective and safe alternatives in the treatment of cancer, researchers are constantly trying to develop new anti-cancer drugs, including those based on compounds of natural origin. Despite huge advances in the pharmaceutical industry, still a vast majority of antibiotics and anti-cancer drugs is derived from natural products [27]. In addition, attention is paid to the usefulness of plant-derived compounds, also from ITC group, serving as additives supporting the therapy with conventional anti-cancer drugs [8]. Taking into account that neoplasms of the hematopoietic system, notably lymphomas, are one of the most frequently diagnosed cancers in dogs [3] and, at the same time, they are the type of cancer for which chemotherapy is the treatment of choice [5], we found it beneficial to test the anti-cancer activity of one of such compounds belonging to ITCs against these cancers. BITC was chosen because its anti-cancer efficacy is considered to be stronger than that of the other members of the ITC family [28]. To our knowledge, this is the first study confirming the cytotoxic and pro-apoptotic activity of BITC against the cancers occurring in dogs. The anti-cancer activity of BITC and other ITCs has been investigated extensively in human in vitro cancer models. For example, BITC was shown to inhibit cell proliferation and growth of HeLa cells [29], human lung large cell carcinoma cells [30], human pancreatic cancer cells [31], and human leukemia HL60 cells [17,18]. It was also shown to reduce the viability of melanoma cells [32], osteosarcoma cells [33], and human gastric adenocarcinoma cells [34]. Moreover, the activity of BITC was confirmed in preclinical in vivo models, where the compound was capable of suppressing pancreatic tumor xenograft growth in immunocompromised mice without any serious side effects [35]. In the presented study, we managed to demonstrate that BITC was active against canine hematopoietic cancer cells. The cancer cell lines most susceptible to BITC were those derived from lymphocytes B, which were killed by the agent at the concentrations only slightly harmful for normal canine cells. This result is consistent with the observations from other studies indicating this type of cancer cells as the most sensitive to anti-cancer therapy [7]. An additional aspect that emerged from the research was different sensitivity of cell lines identified as noncancerous. To compare the activity of the tested compound, we used the established cell lines determined as “noncancerous” i.e., not derived from cancer tissue: MDCK–epithelial cells derived from normal canine kidney [36] and NIH-3T3 -murine embryo fibroblasts, as well as primary cultures of peripheral blood mononuclear cells (PBMCs). Both models have their limitations. The established cell lines ensure consistency and repeatability of results but due to genetic alternations required for immortalization they may not fully reflect the behavior of normal cells [37]. Despite this, they became a common model of healthy cells used for testing drug activity and comparing the activity against cancerous and noncancerous cells, also in the case of BITC [32,35]. Due to a lack of more adequate immortalized canine cell lines, we decided to use MDCK cell line. Additionally, we tested murine embryo fibroblasts, commonly employed for investigating cytotoxic activity of new potential anti-cancer drugs [38,39,40], to enable comparison of the activity of BITC to the activity of other compounds. Primary cell cultures, such as PBMCs, apart from ethical concerns, are burdened with reduced viability and a variable response to stimuli due to the stress induced by preanalytical procedures and host variety. These factors may lead to biased and varied results [41]. On the other hand, these cells represent the type of tissue that appears more appropriate for comparing the activity of a compound with activity on lymphoma and leukemia cells. Higher sensitivity of PBMCs than of the immortalized cell lines may be explained by different activity of the compound toward different cell types but also by the factors discussed above. Nevertheless, our results clearly indicated, a particular selectivity of BITC against cancer cells of B-cell origin. Taking into account, that B-cell lymphomas are the most frequent type of hematopoietic malignances [3], this finding confirmed potential usefulness of BITC in lymphoma and leukemia treatment in dogs.

Previous studies revealed that ITCs cause cell death by caspase-dependent apoptosis [9]. This type of cell death is the most desirable effect of drugs used in cancer treatment [42], and was also observed as the mechanism of anti-cancer activity of BITC [18]. One of the key events during the process is the activation of the effector caspases 3 and 7, which are proteases responsible for the cleavage of proteins, DNA condensation and other characteristic hallmarks of apoptosis [43,44]. Nevertheless, in some circumstances or in the case of disturbances in the cellular apoptotic machinery, apoptosis may occur without activation of caspases [45]. However, we were able to detect the activation of caspase 3/7 in CLB70 and CLBL-1 cell lines treated with BITC and confirmed that caspase activation was essential in the apoptosis induced by the agent, as their inhibition resulted in a partial reduction (in CLBL-1 cell line) or a complete suppression (in CLB70 cell line) of the process. The presented study also confirmed a rapid pro-apoptotic effect of the compound in CLB70 and CLBL-1 cells. Significant increase in the number of cells with active caspase 3/7 was already apparent after three hours of incubation with the compound. After 16 h of incubation, both Annexin V-FITC/PI and active caspase/Sytox staining revealed that the cells were in the late apoptosis. This observation is consistent with the results of a previous study by Zang et al. indicating a swift response of the cell to the compound activity, although in their study the three hour incubation with the compound led to an instant activation of caspases 8 and 9, while caspase 3 activation was delayed for the next three hours [17].

Although caspase 8 is considered to be mainly involved in the extrinsic pathway of apoptosis [46], its activation was also noted during apoptosis prompted by some anti-cancer agents e.g., cisplatin [47]. It was also observed after ITC treatment in human leukemia cells [17] and in human prostate carcinoma cells, where it was shown to be indispensable in the apoptosis [48]. In our study, we also confirmed the activation of caspase 8 in canine lymphoma and leukemia cells exposed to BITC, although the percentage of cells with active caspase 8 was lower than those with activated caspase 3/7.

One of the postulated mechanisms of anti-cancer activity of ITCs is their ability to induce oxidative stress in cancer cells [9]. In physiological conditions, ROS are generated in the cell during various aerobic metabolic processes as by-products, although they are also implicated in cell signaling [49]. The redox balance is maintained due to the action of natural antioxidants, such as e.g., glutathione [50]. Agents capable of increasing ROS formation or targeting glutathione and depleting its levels enhance the amount of ROS in the cell [51]. ROS accumulation constitutes a part of the mechanism of action of many widely used anti-cancer agents [52] as well as some currently investigated natural compounds with potential anti-tumor activity including ITCs [9,53,54]. BITC was shown to induce ROS accumulation in human lung cancer cells [30], human osteosarcoma [33], human melanoma cells [32] and human gastric adenocarcinoma cells [34]. In our study, we also observed increased ROS levels in canine cancer cells derived from lymphocytes B after treatment with BITC. Although this response was not too conspicuous and only short-lived, it might be sufficient to contribute to the deleterious effects exerted by this compound in the examined cell lines. Excessive concentration of ROS in the cell causes oxidative damage of cellular components, such as DNA, proteins and membrane lipids, which results in the initiation of apoptosis [53]. In addition, cancer cells, due to higher metabolic activity and proliferation rate, have higher basal ROS production than normal cells, and are therefore more vulnerable to an additional increase in their formation or disturbance in their ROS scavenging capacity [20,51]. Preincubation with NAC inhibited apoptosis in the cells exposed to BITC. NAC serves as a precursor of glutathione [20], the major factor responsible for maintaining reduced environment in the cell [55]. Its protective role against BITC and other ITC activity is well documented [20,32,33,56], and arises from the fact that increased glutathione level in the cells pretreated with NAC enables them to maintain redox balance and prevent oxidative damage after treatment with agents disabling cell antioxidant system such as ITCs [20]. In addition, previous studies showing that only thiol containing redox compounds such as e.g., NAC were capable of preventing cell death, while free radical scavengers were not, also suggest that the redox stress associated with glutathione depletion, and not direct ROS generation by BITC, is the major mechanism responsible for the cytotoxic effect of ITCs [56,57]. Moreover, the protective role of elevated glutathione levels may result from its direct reaction with BITC that reduces its binding to nucleophilic amino acid residues of cellular proteins [56]. In the case of the presented research, it is not clear what exactly was responsible for the protective effect of increasing the level of glutathione in the cells. The slight and short-term increase in the concentration of free radicals may suggest that direct BITC binding by glutathione and preventing its reaction with other macromolecules in the cell also played a role. However, ROS accumulation might still be involved in BITC activity against the studied canine cell lines i.e., in generating oxidative DNA damage. A suggestion that free radicals participate in BITC influence on the structure of nuclear DNA comes from the research of Yeh Y et al. [56] showing that treatment with BITC led to the formation of hydroxy-20-deoxyguanosine, a marker of oxidative DNA damage. In our study, we also detected DNA damage in CLBL-1 and CLB70 cells exposed to BITC. An increase in H2AX phosphorylation was noted as soon as 1.5 h after the exposure, which was in accordance with the rapid generation of free radicals, the activation of caspases, and the onset of apoptosis under the influence of the compound. Despite the confirmed role of oxidative stress in DNA damage caused by BITC, other mechanisms may also be involved. For example, ITCs were shown to modify cysteine residues on Topoisomerase II α and to induce Topoisomerase II α-mediated DNA cleavage [58]. Nevertheless, taking into account that DNA repair ability of cancer cells is often impaired, it may additionally make them more sensitive to DNA damage generated by the compound and apoptosis [52]. Together with high levels of oxidative stress in cancer cells discussed previously, this may explain a much higher sensitivity of canine cancer cells to BITC observed in our study.

## 4. Materials and Methods

### 4.1. Cell Lines and Cell Culture

The study involved a panel of canine hematopoietic cancer cell lines: CLBL-1 (B-cell lymphoma), GL-1 (B/T-cell leukemia), CLB70 (B-cell chronic lymphocytic leukemia), CNK-89 (NK-cell lymphoma) and noncancerous cell lines: MDCK (Madin-Darby Canine Kidney) and NIH 3T3 (mouse embryonic fibroblasts). MDCK cell line was bought from Sigma-Aldrich (Steinheim, Germany) and NIH-3T3 cell line was acquired from the American Type Culture Collection (ATCC, Rockville, MD, USA). CLBL-1 [59] was a kind gift from Barbara C. Ruetgen from the Institute of Immunology, Department of Pathobiology, University of Veterinary Medicine, Vienna, Austria. GL-1 [60] was provided by Yasuhito Fujino and Hajime Tsujimoto from the University of Tokyo, Department of Veterinary Internal Medicine. CLB70 [61] and CNK-89 [62] were established in our laboratory.

The cell lines were cultured in RPMI 1640 (Institute of Immunology and Experimental Therapy, Polish Academy of Sciences, Wrocław, Poland) or advanced RPMI (Gibco, Grand Island, NY, USA) culture medium supplemented with 2 mM L-glutamine (Sigma Aldrich, Steinheim, Germany), 100 U/mL penicillin, 100 μg/mL streptomycin (Sigma Aldrich, Steinheim, Germany), and 10–20% heat-inactivated fetal bovine serum (Gibco, Grand Island, NY, USA) at 37 °C in a humidified atmosphere containing 5% CO_2_.

### 4.2. Isolation of Peripheral Blood Mononuclear Cells from Healthy Donors

Peripheral blood samples from five dogs of different breed, age and sex, left after routine veterinary diagnostic procedures were used for the isolation of peripheral blood mononuclear cells (PBMCs). The PBMCs were separated by density-gradient centrifugation using Histopaque-1077. Then the PBMC containing layer was collected, the remaining erythrocytes were lysed with 0.84% ammonium chloride, and after two washing steps the PBMCs were resuspended in a complete RPMI medium.

### 4.3. Chemicals and Reagents

BITC was obtained from Sigma-Aldrich (Steinheim, Germany) and dissolved in DMSO (Sigma Aldrich, Steinheim, Germany) to a final concentration of 100 mM immediately prior to the experiments. Histopaque-1077, propidium iodide (PI), ribonuclease A, N-acetyl-l-cysteine (NAC), 2′,7′-Dichlorofluorescin Diacetate (DCF-DA), RIPA buffer and SigmaFAST Protease Inhibitor Cocktail were purchased from Sigma-Aldrich (Steinheim, Germany). Annexin V-FITC was purchased from Immunostep (Salamanca, Spain), CellEvent^®^Caspase-3/7 Green Flow Cytometry Assay and an ECL blotting detection system Kit from ThermoFisher (Waltham, MA, USA), CaspGLOW™ Fluorescein Active Caspase-8 Staining Kit from BioVision (Milpitas, CA, USA), and z-VAD-fmk from InvivoGen (San Diego, CA, USA). Anti-γH2A.X (ab26350) antibody was from Abcam (Cambridge, UK), while anti-β actin (C-4) antibody was from Santa Cruz Biotechnology (Santa Cruz, CA, USA). Anti-mouse/HRP was bought from Dako (Glostrup, Denmark).

### 4.4. Cytotoxicity Assay

The cells plated at the density of 1 × 10^5^ cells per well (canine cancer cell lines) in 96-well plates (Thermo Fisher Scientific, Roskilde, Denmark) or 4 × 10^3^ cells per well (MDCK and NIH 3T3 cell lines) in 24-well plates (Thermo Fisher Scientific, Denmark) were exposed to increasing concentrations (1.25, 2.5, 5, 10, and 20 µM) of BITC for 24 h (DMSO concentration never exceeded 0.4%, which is considered harmless to the cells). In the case of PBMCs, due to limited amount of material available for analysis, the cells were incubated with only two concentrations of the drug: 5 µM and 20 µM. The control cells were incubated in the same conditions with the medium alone. Subsequently, the cells were harvested, washed twice with PBS, stained with PI (final PI concentration 1 µg/mL) and analyzed immediately using a flow cytometer (FACS Calibur; Becton Dickinson, Biosciences, San Jose, CA, USA) and CellQuest 3.lf. software (Becton Dickinson, San Jose, CA, USA). Based on our observations, the concentration that decreased the viability of cells to 50% (EC_50_) was calculated and the concentrations of the compound for the next tests were chosen.

### 4.5. Quantification of Apoptosis

To assess the level of apoptosis induced by BITC, CLB70 and CLBL-1 cells were seeded at a density of 1 × 10^5^ per well in 96-well plates (TPP, Trasadingen, Switzerland) and treated with BITC at the concentration of 4 µM (approximately EC_50_ value for both cell lines) or incubated with the medium alone for 16 h. To evaluate caspase involvement in apoptosis, the cells were preincubated for 1 h with 30 µM of pan-caspase inhibitor (z-VAD-fmk), and to assess the contribution of ROS accumulation, the cells were preincubated for 4 h with 5 mM NAC. Then the cells were collected, and after two washing steps, suspended in a binding buffer and stained with Annexin V-FITC for 10 min at room temperature. Next, PI was added and flow cytometric analysis was immediately performed using a flow cytometer (FACS Calibur; Becton Dickinson, Biosciences, San Jose, USA). CellQuest 3.lf. software (Becton Dickinson, San Jose, CA, USA) was used for data analysis.

### 4.6. Determination of Caspase Activity

To evaluate the level of caspase 3/7 activation, CLB70 and CLBL-1 cells at a density of 1 × 10^5^ per well in 96-well plates (TPP, Trasadingen, Switzerland) were incubated with the medium alone or with BITC (4 µM) in the absence or presence of pan-caspase inhibitor z-VAD-fmk for 1.5, 3 or 16 h. Then, the cells were harvested, washed twice with PBS, and stained with CellEvent^®^Caspase-3/7 Green Detection Reagent according to the manufacturer’s instructions and, after 20 min, SYTOX^®^AADvanced™ dead stain solution was added to the samples for the additional 10 min. For the assessment of caspase 8 activation, after incubation in the same conditions for 16 h, harvesting and washing twice in PBS, FITC-IETD-fmk were added to the samples and the cells were incubated for 1 h. Then they were washed twice and resuspended in a wash buffer. Flow cytometric analysis was performed using a flow cytometer (FACS Calibur; Becton Dickinson, Biosciences, San Jose, CA, USA). CellQuest 3.lf. software (Becton Dickinson, San Jose, CA, USA) was used for data analysis.

### 4.7. Western Blot Analysis of γH2AX Level

CLB70 and CLBL-1 cells seeded in 25 cm^2^ cell culture flasks (TPP, Trasadingen, Switzerland) at the density of 1 × 10^7^ per flask were treated with BITC at the concentration of 4 or 6 µM for 0.5, 1.5 and 3 h or incubated in the medium alone (control). After the desired time, the cells were collected, washed twice with ice-cold PBS, suspended in an ice-cold RIPA buffer supplemented with SigmaFAST Protease Inhibitor Cocktail, and incubated for 20 min on ice. Next, after centrifuging at 10,000 rpm at 4 °C for 12 min, sodium dodecyl sulfate (SDS) sample buffer was added to the suspensions to clear the supernatants and the samples were boiled at 95 °C for 5 min. Then, the samples were subjected to SDS-PAGE on 12% gel (SDS-PAGE running buffer: 25 mM Tris, 192 mM glycine, 0.1% SDS). The resolved proteins were transferred onto PVDF membrane (Millipore, Billerica, MA, USA), using Semidry Transfer Cell (Bio-Rad, Hercules, CA, USA). After transfer, the membrane was blocked with 1% casein in TBS at 4 °C overnight and then incubated with anti-γH2A.X antibody (dilution 1:2000) at room temperature for 1 h, followed by secondary horseradish peroxidase-labeled antibody. The bound antibodies were visualized using ChemiDoc Touch Instruments (BioRad, Hercules, CA, USA).

### 4.8. ROS Detection

For evaluation of ROS accumulation, CLB70 and CLBL-1 cells (1 × 10^5^ per well) were incubated in 96-well plates (TPP, Trasadingen, Switzerland) with BITC at the concentration of 4 or 6 µM for 0.5 or 1.5 h. Next, the cells were harvested, washed twice with PBS and incubated for 1 h with DCF-DA. Then, after washing, the cells were suspended in PBS and analyzed immediately using a flow cytometer (FACS Calibur; Becton Dickinson, Biosciences, San Jose, CA, USA) and CellQuest 3.lf. software (Becton Dickinson, San Jose, CA, USA).

### 4.9. Statistical Analysis

All data were shown as means with standard deviation (SD). Statistical differences were analyzed using Student’s *t*-test for normally distributed values, and one-way ANOVA followed by Tukey’s *post hoc* test were used to test statistical differences among the treatment groups. Statistical analysis was performed with STATISTICA version 13.3 software (TIBCO Software Inc., Palo Alto, CA, USA). The results were considered significant at *p* < 0.05.

## 5. Conclusions

In summary, our results confirmed BITC activity against hematopoietic cancer cells in dogs. The cells derived from B lymphocytes, were particularly sensitive, as their viability was significantly reduced by BITC at a concentration representing only minor toxicity toward normal cells. We demonstrated that the treatment of canine lymphoma and leukemia cells with BITC caused a transient increase in ROS concentration in the cells and DNA damage, which led to caspase-dependent apoptosis. This mode of action was previously confirmed for various types of human neoplasms and is responsible for selective activity toward cancer cells due to their high level of oxidative stress and impaired DNA damage response. Nevertheless, our research shows for the first time that BITC has a similar effect on canine lymphoma and leukemia cells. These findings show that BITC could be potentially useful in anti-cancer therapies in dogs and provide the basis for further analysis of BITC activity against canine cancers.

## 6. Patents

This section is not mandatory but may be added if there are patents resulting from the work reported in this manuscript.

## Figures and Tables

**Table 1:** EC_50_ values for BITC in four canine cancer cell lines after 24 h incubation. The values are means ± SD of three independent experiments. ^a,b,c^ Values with different letters in the superscript differ statistically (*p* < 0.05).

Cell Line	CLBL-1	CLB-70	GL-1	CNK-89
**EC_50_ (µM)**	3.63 ± 0.21 ^a^	3.78 ± 0.25 ^a^	11.14 ± 1.31 ^b^	13.33 ± 0.93 ^c^

## Data Availability

The data supporting the results of this study can be found in the Supplementary Materials of this article and can be obtained on request from the corresponding author.

## Supplementary Materials

The following are available online at https://www.mdpi.com/article/10.3390/ijms222111772/s1, Figure S1: Inhibition of caspase 3/7 activity in CLB70 and CLBL-1 cells treated with BITC. Table S1: Concentration dependent cytotoxic effect of BITC on the canine cancer and noncancerous cell lines. Table S2: Comparison of the BITC effect on canine hematopoietic cancer cell lines and PBMCs. Table S3: Percentage of apoptotic and necrotic CLB70 and CLBL-1 cells incubated with BITC. Table S4: Percentage of cells with active caspase 3/7 after 1.5, 3 and 16 h incubation with BITC. Table S5: Percentage of cells with active caspase 8 incubated with BITC. Table S6: Percentage of cells with active caspase 3/7 after incubation with BITC with or without pretreatment with pancaspase inhibitor. Table S7 Increase in ROS accumulation in CLB70 and CLBL-1 cells incubated with BITC.
