# Supplementary material for: Benzyl Isothiocyanate, a Vegetable-Derived Compound, Induces Apoptosis via ROS Accumulation and DNA Damage in Canine Lymphoma and Leukemia Cells
Source: Int J Mol Sci. 2021 Oct 29;22(21):11772. doi: 10.3390/ijms222111772 (PMC8583731; doi:10.3390/ijms222111772)
Supplement: Supplementary file 1 [file ijms-22-11772-s001.zip › Supplementary files/Supplementary materials.pdf]

A

BITC (µM)/ cell line	0	1.25	2.5	5	10	20
**CLBL-1 (%)**	92.9 ± 3.44	90.5 ± 2.88	69.3 ± 3.18	26.23 ± 4.68	10.91 ± 3.91	7.29 ± 3.65
**CLB70 (%)**	88.5 ± 1.93	86.2 ± 1.47	65.76 ± 2.06	33.63 ± 8.26	16.26 ± 7.48	8.52 ± 1.22
**GL-1 (%)**	92.03 ±2.85	92 ± 2.69	88.9 ± 4.03	79.56 ± 3.41	54.86 ± 6.03	16.53 4.35
**CNK-89 (%)**	94.3 ± 1.13	93.5 ± 1.39	92.43 ± 1.44	86.03 ± 1.66	69.57 ± 5.70	10.95 ± 5.04

B

BITC (µM)/ cell line	0	5	10	20
**MDCK (%)**	93.93 ± 2.29	92.47 ± 1.65	88.80 ± 2.76	81.07 ± 2.80
**3T3 (%)**	97.36 ± 0.76	96.3 ± 0.66	95.36 ± 1.47	93.76 ± 2.34

Table S1. Concentration-dependent cytotoxic effect on the canine cancer (A) and noncancerous cell lines (B). Percentage of living cells after 24 h incubation with benzyl isothiocyanate (BITC) were measured in flow cytometry after staining with propidium iodide (PI).

BITC (µM)/ cell line	5 µM	20 µM
**PBMCs (%)**	91.00 ± 11.14	39.16 ± 10.24
**CLBL-1 (%)**	28.14 ± 4.06	7.76 ± 3.71
**CLB70 (%)**	38.11 ± 9.97	9.65 ± 1.58
**GL-1 (%)**	86.44 ± 1.58	18.07 ± 5.32
**CNK-89 (%)**	91.23 ± 1.15	11.57 ± 5.24

Table S2. Comparison of the BITC effect on canine hematopoietic cancer cell lines and canine peripheral blood mononuclear cells(PBMCs) . Viability of cells after 24 h incubation with 5 µM or 20 µM BITC was measured in flow cytometry after staining with propidium iodide (PI). Percentage of living cells was defined in relations to untreated control cells.

**CLB70**	**Control**	**z-VAD-fmk**	**NAC**	**BITC**	**BITC + z-VAD-fmk**	**BITC + NAC**
**AN+/PI- and AN+/PI+ (%)**	11.33 ± 2.51	8.44 ± 2.97	12.41 ± 0.64	42.056 ± 7.44	11.61 ± 3.48	12.96 ± 1.47
**AN-/PI+ (%)**	1.41 ±0.57	1.58 ± 0.68	1.19 ± 0.69	5.80 ± 2.62	2.87 ± 1.69	1.84 ± 0.17

**CLBL-1**	**Control**	**z-VAD-fmk**	**NAC**	**BITC**	**BITC + z-VAD-fmk**	**BITC + NAC**
AN+/PI- and AN+/PI+ (%)	6.62 ± 1.01	6.2 ± 1.10	5.94 ± 0.56	39.75 ± 7.25	21.94 ± 3.98	6.78 ± 0.32
AN-/PI+ (%)	1 ± 0.31	1.17 ± 0.27	0.76 ± 0.24	4.57 ± 1.85	4.74 ± 2.59	0.86 ± 0.10

Table S3. Percentage of apoptotic and necrotic CLB70 and CLBL-1 cells after 16 h incubation with BITC. Cells were stained with Annexin V-FITC/PI and the analysed in flow cytometry.

BITC (4 µM) /cell line	Control	1.5h	3h	16h
**CLB-70 (%)**	6.64 ± 1.76	7.26 ± 1.06	14.56 ± 3.55	35.77 ± 8.26
**CLBL-1 (%)**	7.28 ± 3.26	9.37 ± 2.95	18.09 ± 5.31	35.72 ± 2.76

Table S4. Percentage of cells with active caspase 3/7 after 1.5, 3 and 16 h incubation with BITC. Cells were stained with CellEvent®Caspase-3/7 Green Detection Reagent and SYTOX®AADvanced™ and analysed in flow cytometry.

	**Control**	**BITC 4 µM**
**CLB70 (%)**	6.13 ± 3.46	25.77 ± 5.59
**CLBL-1 (%)**	4.13 ± 3.33	27.10 ± 3.73

Table S5. Percentage of cells with active caspase 8 after 16 h incubation with BITC. Cells were stained with FITC-IETD-fmk and analysed in flow cytometry.

CLB70	BITC 0 µM	BITC 4 µM
**Control (%)**	5.03 ± 2.20	35.77 ± 8.26
**z-VAD-fmk (%)**	3.84 ± 2.63	6.35 ± 3.56

CLBL-1	BITC 0 µM	BITC 4 µM
**Control (%)**	4.12 ± 1.44	35.72 ± 2.76
**z-VAD-fmk (%)**	3.30 ± 0.58	25.68 ± 3.07

Table S6. Percentage of cells with active caspase 3/7 16 h incubation with BITC with (z-VAD-fmk) or without (Control) pretreatemnt with pancaspase inhibitor. Cells were stained with CellEvent®Caspase-3/7 Green Detection Reagent and SYTOX®AADvanced™ and analysed in flow cytometry

CLB70	Control	BITC 4 μM	BITC 6 μM
**0.5 h**	100.00	144.36 ± 10.89	135.79 ± 18.20
**1.5 h**	100.00	108.69 ± 6.12	101.95 ± 8.43

CLBL-1	Control	BITC 4 μM	BITC 6 μM
**0.5h**	100.00	152.33 ± 13.40	147.32 ± 11.37
**1.5h**	100.00	119.02 ± 1.98	113.10 ± 16.93

Table S7. Increase (%) in ROS accumulation in CLB70 and CLBL-1 cells incubated with BITC for 0.5 and 1.5 h. Cells stained with 2′,7′-Dichlorofluorescin Diacetate (DCF-DA) were analysed in flow cytometry. The fluorescence in untreated cells (Control) was defined as 100%.
