# Supplementary figures and images for: Benzyl Isothiocyanate, a Vegetable-Derived Compound, Induces Apoptosis via ROS Accumulation and DNA Damage in Canine Lymphoma and Leukemia Cells
Source: Int J Mol Sci. 2021 Oct 29;22(21):11772. doi: 10.3390/ijms222111772 (PMC8583731; doi:10.3390/ijms222111772)

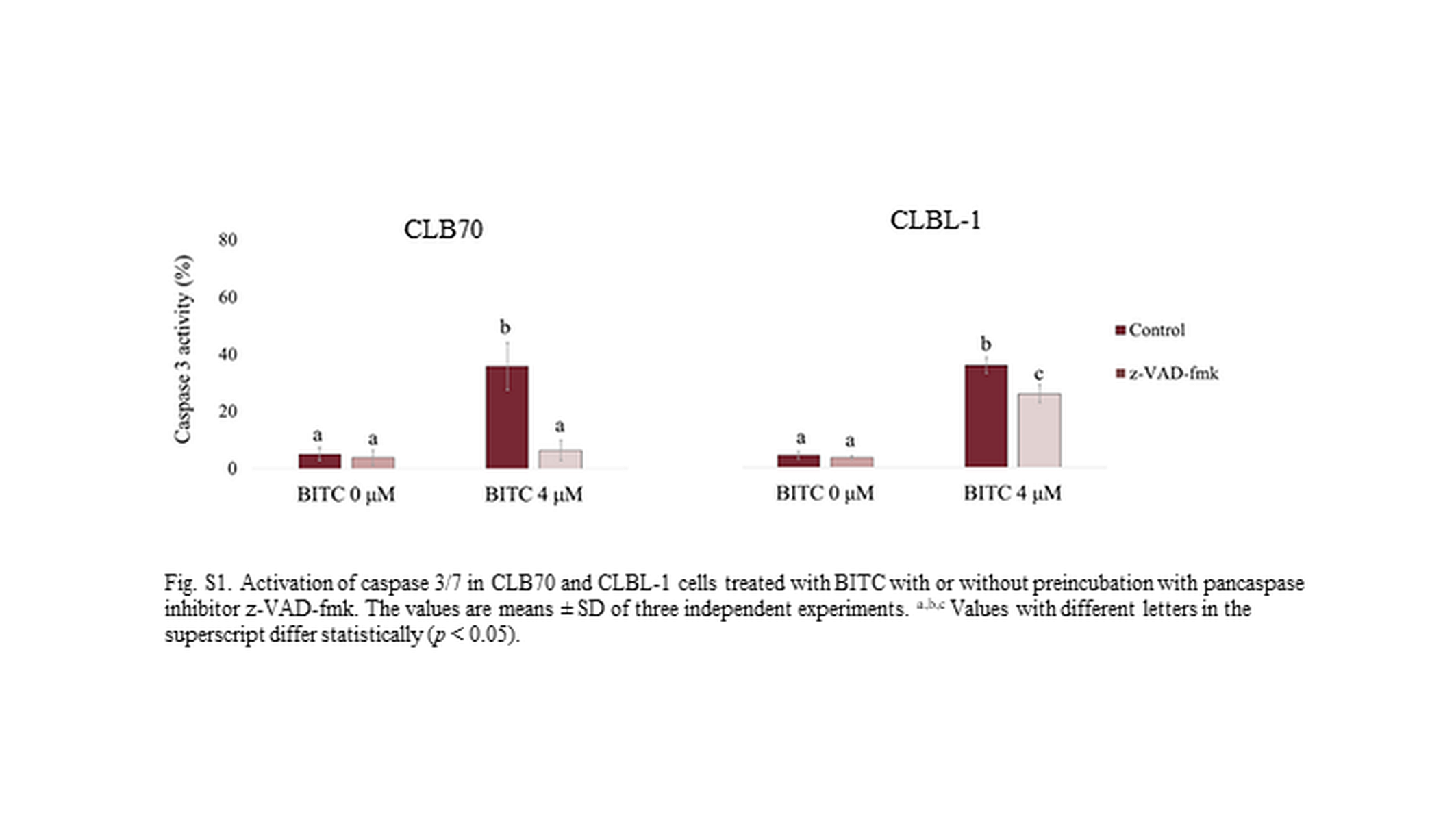

Supplement: Supplementary file 1 [file ijms-22-11772-s001.zip › Supplementary files/Fig. S1.tif]
